# Improving cervical screening risk stratification through extended human papillomavirus genotyping and age-specific data

**DOI:** 10.1093/eurpub/ckag139

**Published:** 2026-08-17

**Authors:** Aarno Leino, Eero Numminen, Veronika Fonagy, Minna Paloniemi, Tapio Seiskari, Ivana Kholová, Saara Kares, Karolina Louvanto

**Affiliations:** Department of Obstetrics and Gynecology, Faculty of Medicine and Health Technology, Tampere University, Tampere, Finland; Department of Obstetrics and Gynecology, Faculty of Medicine and Health Technology, Tampere University, Tampere, Finland; Department of Microbiology, Fimlab Laboratories, Tampere, Finland; Department of Microbiology, Fimlab Laboratories, Tampere, Finland; Faculty of Medicine and Health Technology, Tampere University, Tampere, Finland; Department of Microbiology, Fimlab Laboratories, Tampere, Finland; Department of Pathology, Fimlab Laboratories, Tampere, Finland; Department of Pathology, Faculty of Medicine and Health Technology, Tampere University, Tampere, Finland; Department of Pathology, Fimlab Laboratories, Tampere, Finland; Department of Obstetrics and Gynecology, Faculty of Medicine and Health Technology, Tampere University, Tampere, Finland; Department of Obstetrics and Gynecology, Tampere University Hospital, Tampere, Finland

## Abstract

High-risk human papillomavirus (HR-HPV) screening effectively reduces cervical cancer incidence, but limited specificity leads to excessive colposcopy referrals. Extended HR-HPV genotyping improves triage by accounting for genotype-specific oncogenic risks, offering a more precise framework for patient management than traditional pooled testing. This study analysed real-world screening data from a Finnish cohort of 2368 HR-HPV-positive women (Tampere region, 2017–2019) with up to 6.5 years of follow-up. Extended genotyping was performed using the Seegene Anyplex™II HPV28 Detection assay. Genotype-specific prevalence and cumulative incidence of histology-confirmed High-grade Squamous Intraepithelial Lesions or worse (HSIL+) were assessed and stratified by reflex cytology. Among 3099 detected HR-HPV genotypes, HPV16 was most prevalent (14.3%) and carried the highest HSIL+ risk (35.7%), followed by HPV33 (24.6%) and HPV18 (20.8%). A clear risk hierarchy emerged: the lowest-risk group (HPV59/39/68/51/56/66) had an HSIL+ incidence below 4.2%. Notably, HPV16-positive women with NILM/ASC-US cytology faced a 23.9% HSIL+ risk, exceeding the 21.1% risk seen in the lowest-risk genotype group even when accompanied by LSIL+ cytology. For those with both NILM/ASC-US and the lowest-risk genotypes, HSIL+ incidence was only 3.4%. A distinct hierarchy of oncogenic risk exists within this population-based cohort. Incorporating genotype-specific data into national screening algorithms is strongly supported. This allows for immediate colposcopy for high-risk groups (e.g. HPV16 with mild cytology) while justifying extended follow-up for lower-risk types, optimizing clinical resources and reducing unnecessary procedures.

## Introduction

At the beginning of twenty-first century, human papillomavirus (HPV) testing effectively displaced the conventional cervical cytology as the primary modality for cervical cancer screening, primarly due to its superior sensitivity in detecting precancers and cancers [1]. However, the trade-off for this high sensitivity is a relatively low specificity, leading to clinical challenges such as excessive follow-up procedures and increased colposcopy referrals among screened women [2]. To improve the specificity and manage the resulting burden, reflex cytology has been adopted for the risk stratification of the high-risk (HR)-HPV-positive women [3]. Yet, reflex cytology alone often fails to provide the detailed risk assessment needed due to its subjective nature, limited reproducibility, and inherent inability to capture underlying oncogenic potential when cytological abnormalities are absent or minimal, making it difficult to accurately distinguish individuals at immediate risk, high-risk from those at low risk of developing cervical cancer [4].

The complexity of risk is rooted in the viral diversity. The International Agency for Research on Cancer (IARC) classifies 12 types as carcinogenic to humans, with two additional types (probably carcinogenic and possibly carcinogenic) which are usually included in commercial screening panels due to their associated risk [5]. Crucially, even among these IARC-classified high-risk types, there is a vast disparity in the risk of an infection progressing to invasive cervical cancer. The global burden is overwhelmingly driven by HPV16 and HPV18, which account for nearly 80% of all invasive cervical cancer cases [6]. The remaining 12 HR-HPV types combined contribute a significantly smaller proportion, highlighting the substantial difference between simple viral prevalence and true oncogenic potential among high-risk strains.

To refine the triage process and utilize the disparity of different HR-HPV types, the use of extended HPV genotyping has been introduced [7]. By identifying the specific HR-HPV genotypes, clinicians can implement strategies that prioritize women based on their markedly different, genotype-specific risks of cervical cancer and precancerous lesions [5], thus significantly improving the effectiveness and efficiency of screening programs. Recent studies have demonstrated this benefit, showing that combining extended genotyping with cytology can reduce overtreatment and better identify the women at greatest risk [8].

Although a growing body of literature assesses the risks of individual HR-HPV genotypes, more real-world data and systemic validation are needed. Critically, the impact of adapting new triage strategies depends on a thorough understanding of the population-level prevalences and associated risks of different HR-HPV genotypes [9].

Currently in Finland all HR-HPV genotypes are managed with the same level of urgency, with triage decisions based solely on cytology [10]. To update the screening algorithm in Finland, we utilized our population-based screening data to evaluate the prevalences and risks for histology confirmed high-grade squamous intraepithelial lesion or worse (HSIL+) among different HR-HPV genotypes.

## Methods

### Study cohort

The study utilized data from 34 871 women who participated in the Finnish national cervical cancer screening programme in the city of Tampere and the surrounding municipalities (Kangasala, Lempäälä, Nokia, Pirkkala and Ylöjärvi) between 2017 and 2019. The program is coordinated by the publicly owned private healthcare provider, Fimlab Laboratories Ltd. This analysis specifically focused on the 2368 women who were initially determined to be HR-HPV-positive, with a follow-up period between 4.5 and 6.5 years extending until the end of 2023. In Finland, cervical cancer screening is conducted at 5-year intervals for women aged 30–60 years old (the screening age was extended to include women up to 65 years old from 2021 onwards). Given their birth years (1957–1989), the participants in this screening round represent a completely unvaccinated population prior to the 2013 introduction of the national adolescent HPV vaccination program.

Women who tested negative for HR-HPV were considered screening negative and scheduled for routine follow-up screening after 5 years. All HR-HPV-positive participants were managed according to the national triage protocol and triaged with cervical cytology. Women with cytological findings of low-grade squamous intraepithelial lesion or worse (LSIL+), or glandular lesions such as atypical glandular cells not otherwise specified (AGC-NOS) or atypical glandular cells favor neoplasia (AGC-FN) were referred directly to colposcopy. Conversely, those with milder cytology results of atypical squamous cells of undetermined significance (ASC-US) or negative for intraepithelial lesion or malignancy (NILM) were scheduled for repeat screening after 12–24 months [11]. Women who remained HR-HPV-positive upon repeat testing after 12–24 months were referred for colposcopy regardless of cytology findings. Screening test results and clinical follow-up data for all HR-HPV-positive women referred for colposcopy were obtained from patient records at Tampere University Hospital (TAUH), Tampere, Finland.

### Ethics declarations

The study protocol was approved by the Ethical Board of the Pirkanmaa Wellbeing Services County (R13094/25.03.2013 and R21034/31.03.2021). Retesting of the screening sample was approved by the Finnish Medical Agency (Fimea/2021/007586). No individual informed consent was required, as the study was conducted within routine screening practice, and individual participant data were not recognizable.

### HPV genotyping and cervical cytology

Initial screening HPV genotyping was performed using the Abbott RealTime HR-HPV array. The assay detects HPV16 and 18 separately, while grouping the remaining 12 high-risk genotypes together (31, 33, 35, 39, 45, 51, 52, 56, 58, 59, 66, and 68). Cervical cytology among the HR-HPV-positive was classified according to the Bethesda System (TBS) [12].

For the purpose of this study, all initial HR-HPV-positive samples were retested using the Seegene Anyplex™II HPV28 Detection assay, which detects all 14 HR-HPV genotypes (16, 18, 31, 33, 35, 39, 45, 51, 52, 56, 58, 59, 66, and 68) separately, in addition to 14 low-risk HPV genotypes (6, 11, 26, 40, 42, 43, 44, 53, 54, 61, 69, 73, 70, 82).

### Colposcopy referrals and punch biopsies

All colposcopy examinations were conducted at the gynecology outpatient clinic of TAUH, where punch biopsies were obtained for histopathological diagnoses. Women diagnosed with histological HSIL were treated with loop electrosurgical excision procedure (LEEP). In cases where a major discrepancy occurs between referral cytology and colposcopic biopsy findings (e.g. HSIL cytology with negative histology), women are scheduled for a repeat colposcopy assessment within 6 months with repeat biopsies, followed by LEEP if HSIL is confirmed, or annual co-testing (HPV and cytology) for minor findings until HPV clearance. Persistent discrepancies undergo multidisciplinary team review to reevaluate the cytological and histological slides. All patients were subsequently managed in accordance with the Finnish Current Care Guidelines for cervical precancerous lesions and cervical cancer [13].

### Statistical analysis

Of the 2585 retested samples 2368 were used for the analysis. Samples were excluded if they were not primary screening samples (*n* = 7), or if retesting yielded an unreliable result (HR-HPV-negative, *n* = 167; or only low-risk HPV-positive, *n* = 43). Cervical cytology was stratified into NILM/ASC-US and LSIL+ groups. The LSIL+ group included LSIL, atypical squamous cells cannot exclude HSIL (ASC-H), HSIL, AGC-NOS, and AGC-FN. If multiple cytological results were available for a woman, only the most severe result was used.

For the primary endpoint, the most severe histological finding recorded during the follow-up period (up to 6.5 years) was used. HSIL+ histology included cases with HSIL, adenocarcinoma *in situ* (AIS), and carcinomas. HR-HPV-positive women with HPV-negative result at retesting were not eligible for colposcopy and were therefore presumed not to have HSIL+ histology in the analysis.

For the age-based comparison, women were stratified into three age groups based on screening invitation age: the youngest age group: ages 30 and 35; the second group: ages 40 and 45; and the oldest group: ages 50, 55, and 60. The cumulative incidences of histology confirmed HSIL+ were assessed for the 14 different HR-HPV genotypes, both with and without cytology results. Chi-squared test was used to compare these cumulative incidences between different HR-HPV genotypes and between the different age groups. All statistical analyses were conducted using STATA17.0 (STATA Corp., TX, USA), with a two-sided *P-*value of less than .05 considered to indicate statistical significance.

## Results

A total of 34 871 women attended the routine national cervical cancer screening in Tampere and the surrounding regions between 2017 and 2019. Of these, 2578 (7.4%) were initially HR-HPV-positive. Following exclusions, 2368 HR-HPV-positive women (91.9% of the positive cohort) were included for the final analysis using extended genotyping.

The prevalence of the 14 different HR-HPV genotypes showed considerable variation across the cohort (Table 1). A total of 3099 HR-HPV genotypes were detected, highlighting the frequency of multiple infections. The distribution was highly skewed, confirming the dominant oncogenic types. HPV16 was the most prevalent genotype, accounting for 443 (14.3%) cases of all detected genotypes. HPV31 and HPV52 were the next most common, with 344 (11.1%) cases and 304 (9.8%) cases, respectively. In contrast, HPV18 accounted for only 178 (5.7%) cases, while the least prevalent genotypes were HPV35 (118 cases, 3.8%), HPV59 (133 cases, 4.3%), and HPV33 (138 cases, 4.5%).

**Table 1.** Prevalence and distribution of 14 high-risk HPV genotypes^a^ among screening HPV-positive women (*n* = 2368), stratified by age, Tampere region, and surrounding municipalities, Finland (2017–2019)

IARC classification^b^	HPV genotype	30	35	40	45	50	55	60	All
		*n* (%)	*n* (%)	*n* (%)	*n* (%)	*n* (%)	*n* (%)	*n* (%)	*n* (%)
Group 1	HPV16	162 (17.0)	92 (16.6)	53 (13.8)	32 (12.9)	34 (9.3)	40 (12.4)	30 (11.1)	443 (14.3)
	HPV18	66 (6.9)	32 (5.8)	21 (5.5)	16 (6.4)	18 (4.9)	10 (3.1)	15 (5.6)	178 (5.7)
	HPV31	106 (11.1)	71 (12.8)	41 (10.6)	22 (8.8)	48 (13.1)	26 (8.1)	30 (11.1)	344 (11.1)
	HPV33	46 (4.8)	29 (5.2)	11 (2.9)	11 (4.4)	12 (3.3)	21 (6.5)	9 (3.3)	138 (4.5)
	HPV35	22 (2.3)	19 (3.4)	16 (4.2)	9 (3.6)	20 (5.5)	18 (5.6)	14 (5.2)	118 (3.8)
	HPV39	62 (6.5)	33 (6)	30 (7.8)	13 (5.2)	19 (5.2)	15 (4.7)	9 (3.3)	181 (5.8)
	HPV45	70 (7.4)	41 (7.4)	33 (8.6)	24 (9.6)	36 (9.8)	24 (7.5)	31 (11.5)	259 (8.4)
	HPV51	84 (8.8)	35 (6.3)	32 (8.3)	12 (4.8)	29 (7.9)	20 (6.2)	21 (7.8)	233 (7.5)
	HPV52	99 (10.4)	52 (9.4)	45 (11.7)	20 (8)	31 (8.5)	32 (9.9)	25 (9.3)	304 (9.8)
	HPV56	50 (5.3)	32 (5.8)	19 (4.9)	29 (11.6)	27 (7.4)	25 (7.8)	24 (8.9)	206 (6.6)
	HPV58	38 (4.0)	29 (5.2)	16 (4.2)	19 (7.6)	23 (6.3)	20 (6.2)	13 (4.8)	158 (5.1)
	HPV59	41 (4.3)	26 (4.7)	22 (5.7)	6 (2.4)	16 (4.4)	10 (3.1)	12 (4.4)	133 (4.3)
Group 2A	HPV68	64 (6.7)	26 (4.7)	25 (6.5)	17 (6.8)	23 (6.3)	30 (9.3)	16 (5.9)	203 (6.6)
Group 2B	HPV66	42 (4.4)	37 (6.7)	21 (5.5)	19 (7.6)	30 (8.2)	31 (9.6)	21 (7.8)	201 (6.5)
	Any high-risk HPV	952	554	385	249	366	322	270	3099

a Fourteen most oncogenic high-risk HPV defined by International Agency for Research on Cancer (IARC).

b Group 1: carcinogenic to humans (12 types); Group 2A: probably carcinogenic to humans (1 type); and Group 2B: possibly carcinogenic to humans (1 type).

The prevalence of the most common genotype varied between different age groups, with younger women generally showing a higher detection rate of total HR-HPV genotypes. This trend was quantified by the fact that the greatest number of genotypes was found in the youngest age group (30-year-olds, *n* = 952), followed by 35-year-olds (*n* = 544) and 40-year-olds (*n* = 385). The rate of multiple infections peaked in the 30-year-old group with an average of 1.37 HR-HPV genotypes per woman. Crucially, this co-infection density remained remarkably stable across the older age strata, tracking at 1.28 in both 35- and 40-year-olds, 1.22 in 45-year-olds, 1.27 in 50-year-olds, 1.33 in the age group of 55-year-olds, and 1.30 in 60-year-olds.

The cumulative incidence of histology confirmed HSIL+ showed significant variation across the 14 HR-HPV genotypes (*P <* .001), establishing a clear oncogenic risk hierarchy (Table 2). HPV16 conferred the highest risk (35.7% cumulative incidence for HSIL+), followed by HPV33 (24.6%), HPV18 (20.8%), HPV58 (19.6%), HPV31 (18.4%), and HPV35 (16.16%). Conversely, the least oncogenic types like HPV59, HPV68, and HPV39 showed incidence rates below 4.2%, which is substantially lower than the risk for the overall HR-HPV-positive group (17.02%) or the more oncogenic types. The risk also showed a strong age-dependent decline, dropping from 21.4% in the 30–35 age group to 9.5% in the 50–60 age group. This decline was statistically significant (*P < *.05) for the four most common oncogenic types (HPV16, 31, 33, and 52), with the highest cumulative incidence for all four observed in the youngest (30–35 years) stratum. For example, HPV16 incidence fell sharply from 42.5% in the youngest group to 20.2% in the oldest one.

**Table 2.** Cumulative incidence of histology confirmed HSIL+ among the 14 high-risk (HR) HPV genotypes^a^ stratified by age groups of 30–35, 40–45, and 50–60

		**30–35 (*n*** **=** **1126)**	**40–45 (*n*** **=** **504)**	**50–60 (*n*** **=** **738)**	**All (*n*** **=** **2368)**	
HR-HPV genotypes	*n*	Cumulative HSIL+ %	Cumulative HSIL+ %	Cumulative HSIL+ %	Cumulative HSIL+ %	*P*-values*
HPV16	443	42.52	34.12	20.19	35.67	.000
HPV18	154	20.48	31.25	12.82	20.78	.162
HPV31	288	22.76	22.64	8.89	18.40	.019
HPV33	122	35.94	25.00	5.26	24.59	.002
HPV35	99	15.63	23.81	13.04	16.16	.537
HPV39	97	…	8.00	9.09	4.12	.107
HPV45	198	7.06	14.63	5.56	8.08	.211
HPV51	160	8.86	…	7.84	6.88	.250
HPV52	232	21.15	10.71	6.94	14.22	.020
HPV56	128	4.65	8.33	4.08	5.47	.667
HPV58	143	23.33	24.24	12.00	19.58	.245
HPV59	81	4.76	…	4.55	3.70	.661
HPV66	146	1.89	12.12	6.67	6.16	.155
HPV68	77	3.13	9.09	…	3.90	.277
Any HR-HPV	2368	21.40	18.25	9.49	17.02	.000
*P*-values**		.000	.000	.036	.000	

a Accounting each woman only once based on the most oncogenic HPV genotype, either single- or part of multi-infection.

* *P-*value for the differences of cumulative incidence between different age groups.

** *P-*value for the differences of cumulative incidence between the 14 high-risk HPV genotypes.

The cumulative incidence of histology confirmed HSIL+ was calculated for 14 HR-HPV genotypes, stratified by reflex cytology (NILM/ASC-US vs. LSIL+) (Table 3). Reflex cytology provided strong stratification: the overall cumulative incidence of HSIL+ was 48.4% among HR-HPV-positive women with LSIL+ cytology compared to only 11.0% among those with NILM/ASC-US cytology. The highly oncogenic types confirmed this trend across the LSIL+ stratum, HPV16 (68.4%), HPV18 (61.8%), HPV33 (56.5%), and HPV31 (50.0%) all showing HSIL+ incidence rates above 50% when concurrent with LSIL+, and even types like HPV45 showing a substantial risk of 34.8%. For women with NILM/ASC-US cytology, the absolute risk was significantly lower, but the risk hierarchy remained clear: the highest risk was found with HPV16 (23.9%) and HPV33 (17.2%), with risk considerably lower for other high-risk types, such as HPV18 (9.2%) and HPV45 (4.6%).

**Table 3.** Cumulative incidence of histology confirmed HSIL+ for 14 HR-HPV genotypes,^a^ stratified by reflex cytology results (NILM/ASC-US vs. LSIL+)

			30–35 (*n* = 1126)	40–45 (*n* = 504)	50–60 (*n* = 738)	All (*n* = 2367)	
HR-HPV genotypes	Cytology	*n*	Cumulative HSIL+ %	Cumulative HSIL+ %	Cumulative HSIL+ %	Cumulative HSIL+ %	*P*-values*
HPV16	NILM/ASC-US	326	28.98	24.62	12.94	23.93	.017
	LSIL+	117	73.08	65.00	52.63	68.38	.214
HPV18	NILM/ASC-US	119	11.11	9.09	5.88	9.24	.698
	LSIL+	34	50.00	80.00	75.00	61.76	.237
HPV31	NILM/ASC-US	240	15.25	15.91	5.13	12.08	.072
	LSIL+	48	55.56	55.56	33.33	50.00	.411
HPV33	NILM/ASC-US	99	25.00	22.22	3.03	17.17	.030
	LSIL+	23	68.75	50.00	20.00	56.52	.156
HPV35	NILM/ASC-US	81	11.54	11.76	10.53	11.11	.987
	LSIL+	18	33.33	75.00	25.00	38.89	.232
HPV39	NILM/ASC-US	92	…	8.33	9.52	4.35	.110
	LSIL+	5	…	…	…	0.00	–
HPV45	NILM/ASC-US	175	3.80	8.57	3.28	4.57	.444
	LSIL+	23	50.00	50.00	18.18	34.78	.278
HPV51	NILM/ASC-US	133	3.17	…	2.33	2.26	.649
	LSIL+	27	31.25	…	37.50	29.63	.467
HPV52	NILM/ASC-US	210	17.71	10.00	6.25	12.83	.082
	LSIL+	22	62.50	16.67	12.50	31.82	.064
HPV56	NILM/ASC-US	111	5.13	3.33	4.76	4.50	.934
	LSIL+	17	…	33.33	…	11.76	.125
HPV58	NILM/ASC-US	122	16.33	22.22	10.87	15.57	.427
	LSIL+	21	54.55	33.33	25.00	42.86	.507
HPV59	NILM/ASC-US	79	4.76	…	…	2.53	.405
	LSIL+	2	…	…	50.00	50.00	–
HPV66	NILM/ASC-US	128	2.08	4.00	5.45	3.91	.678
	LSIL+	18	…	37.50	20.00	22.22	.283
HPV68	NILM/ASC-US	70	3.23	5.56	…	2.86	.575
	LSIL+	7	…	25.00	…	14.29	.646
Any HR-HPV	NILM/ASC-US	1985	13.73	11.93	6.40	10.98	.000
	LSIL+	382	56.72	49.41	30.21	48.43	.000

a Accounting each woman only once based on the most oncogenic HPV genotype, either single- or part of multi-infection.

* *P-*value for the differences in cumulative incidence between the age groups.

The overall cumulative incidence of HSIL+ was significantly higher in younger age groups for both cytology strata (*P* < .001). The risk for the Any HR-HPV -group with LSIL+ cytology decreased from 56.7% in the 30–35 age group to 30.2% in the 50–60 age group. The risk for the Any HR-HPV -group with NILM/ASC-US cytology similarly decreased from 13.7% to 6.4% across the same age strata. Only HPV16 and 33 with NILM/ASC-US cytology showed statistically significant age-related differences in HSIL+ incidence (*P* < .03), confirming that the risk for HSIL+ decreased with age for these two highly oncogenic types when cytology was normal or mildly abnormal.

To ensure that the observed risk hierarchy was not unduly influenced by multiple infections, the cumulative incidence of HSIL+ for single HR-HPV infections was analysed (Supplementary Table S1). The findings confirmed the overall risk hierarchy established in Table 2. Notably, the overall cumulative incidence for HSIL+ among single infections (14.7%) was slightly lower than that for the entire cohort (17.0%), but the age-dependent decline in risk remained highly significant (*P* = .000). Furthermore, the risk ranking remained consistent: HPV16 retained the highest risk (34.0%) and the least oncogenic types (e.g. HPV59, HPV39) remained below 4.2%. Only HPV18 showed a notable difference, with its age-dependent decline becoming statistically significant for single infections (*P* = .027).

The 14 HR-HPV genotypes were categorized into four distinct groups based on their established oncogenic risk (HPV16; HPV18/45; HPV33/58/31/52/35; and HPV59/39/68/51/56/66) [5] to further assess the cumulative incidence of histology-confirmed HSIL+ (Table 4). The differences in cumulative incidence between these four groups were highly significant (*P* < .001). The highest risk was concentrated in the HPV16 group (35.7%), while the least oncogenic group (HPV59/39/68/51/56/66) showed significantly lowest risk of just 5.4%. The intermediate groups showed the following risks: HPV33/58/31/52/35 (18.1%) and HPV18/45 (13.6%). This ranking shifted slightly when cytology was incorporated: among women with LSIL+ reflex cytology, the HPV18/45 group (50.9%) showed a slightly higher cumulative incidence of HSIL+ than the HPV33/58/31/52/35 group (45.5%). The least oncogenic group, HPV59/39/68/51/56/66, posed a minimal risk for HSIL+ when associated with NILM/ASC-US cytology (3.4%), although the risk rose to 21.1% with LSIL+ cytology.

**Table 4.** Cumulative incidence of histology confirmed HSIL+ of 14 HR-HPV genotypes^a^ grouped by oncogenic risk (HPV16, HPV18/45, HPV33/58/31/52/35, and HPV59/39/68/51/56/66), stratified by age

			**30–35 (*n*** **=** **1126)**	**40–45 (*n*** **=** **504)**	**50–60 (*n*** **=** **738)**	**All (*n*** **=** **2368)**	
HR-HPV genotypes	Cytology	*n*	Cumulative HSIL+ %	Cumulative HSIL+ %	Cumulative HSIL+ %	Cumulative HSIL+ %	*P*-values*
HPV16	–	443	42.52	34.12	20.19	35.67	.000
	NILM/ASCUS	326	28.98	24.62	12.94	23.93	.017
	LSIL+	117	73.08	65.00	52.63	68.38	.214
HPV18/45	–	352	13.69	21.92	8.11	13.64	.028
	NILM/ASCUS	294	7.04	8.77	4.21	6.46	.502
	LSIL+	57	50.00	68.75	33.33	50.88	.142
HPV33/58/31/52/35	–	884	23.95	19.67	9.12	18.10	.000
	NILM/ASCUS	752	17.21	15.38	6.95	13.30	.001
	LSIL+	132	57.35	44.44	24.32	45.45	.005
HPV59/39/68/51/56/66	–	689	4.35	6.75	5.73	5.37	.527
	NILM/ASCUS	613	2.96	3.55	3.96	3.43	.837
	LSIL+	76	17.24	27.27	20.00	21.05	.676
Any HR-HPV	–	2368	21.40	18.25	9.49	17.02	.000
*P*-values**			.000	.000	.000	.000	

a Accounting each woman only once based on the most oncogenic HPV genotype, either single- or part of multi-infection.

* *P-*value for the differences of cumulative incidence between different age groups.

** *P-*value for the differences of cumulative incidence between different HPV genotype -groups.

A significant age-related decline in risk was observed for the HPV16 and HPV33/58/31/52/35 groups (*P* < .001 for both), with the highest cumulative incidences observed in the youngest age group (42.5% and 24.0%, respectively). In contrast, the HPV18/45 group showed its highest incidence (21.9%) in the 40–45-year-old age group, rather than the youngest (13.7%), a difference that was statistically significant (*P* = .028).

## Discussion

Extended HR-HPV genotyping has emerged as a globally relevant tool for refining the management of HR-HPV-positive women in cervical cancer screening programs. Our real-world, population-based data from the Tampere region (2017–2019) strongly support the immediate utility of extended genotyping as a triage method. We demonstrate a clear hierarchy of oncogenic risk and pronounced risk disparity across genotypes and age groups. Moving forward, the varying risk of progression to invasive cancer conferred by specific HPV genotypes must be carefully evaluated to maximize the utility of this genotype information in updated screening algorithms.

Our age-specific prevalence data provide valuable insights for improving screening strategies [9]. The distribution of HR-HPV types in our cohort, with HPV16, HPV31, and HPV52 as the most prevalent genotypes, showed greater similarity to a recent Finnish observational study than to broader global systematic reviews, suggesting regional consistency [6, 14]. Similarly to the ATHENA trial, the prevalence of HR-HPV was highest in the youngest age group and decreased with age [9]. Crucially, despite often being grouped together, the prevalence of HPV16 was substantially higher than HPV18 (14.3% vs. 5.7%), a finding consistent with previous literature [15].

Our study confirms the clinical importance of extended genotyping in risk stratification [8]. HPV16 consistently conferred the highest risk for histology confirmed HSIL+ (35.7%), a finding strongly aligned with previous literature and supporting immediate management strategies [8, 16]. Our full risk ranking (HPV16 > HPV33 > HPV18 > HPV58) closely mirrors the findings of the Swedescreen study [16]. The overall risk for HSIL+ observed in our study, including the HPV16 risk of 35.7% (compared to 38% in other long-term studies), is likely due to our shorter follow-up period [6]. The risk for non-HPV16 HR-HPV types was generally consistent with previous Finnish findings [6]. However, the observed discrepancy for HPV18 (earlier Finnish data had a risk of 14.9% vs. our 20.8% for HSIL+), might be due to temporal shifts or geographical differences influencing the distribution of prevalent HPV18 sublineages, which may possess higher clinical oncogenicity [17, 18]. Similarly, the intermediate-risk categorization of types such as HPV31, 33, 35, 52, and 58 often varies globally, driven primarily by differences in regional HPV prevalence and the specific testing assays utilized.

Our finding that HPV16-positive women with NILM/ASC-US cytology have a high risk of 23.9% for HSIL+ strongly adds to the evidence supporting immediate colposcopy referral for this specific group, even without repeated testing [8, 14, 19]. This is further supported by evidence that CIN2 lesions caused by HPV16 are more likely to represent true precancer [20, 21]. HPV18/45 infections with concurrent NILM/ASC-US cytology showed lower HSIL+ risk, suggesting that immediate colposcopy may not be necessary. However, because these genotypes are more strongly associated with adenocarcinoma [21, 22], our finding that the highest incidence for the HPV18/45 group was in the 40–45 age group (not the youngest) necessitates detailed follow-up consideration and potentially alternative clinical protocols. Because individual classifications for CIN2 and CIN3 were unavailable in most cases due to their combined reporting as HSIL, we could not further evaluate the specific role of CIN2 in HPV18/45 cases separately. In contrast, the lowest-risk group (HPV59/39/68/51/56/66) with concurrent NILM/ASC-US cytology comprised 25.9% of the cohort and demonstrated an extremely low HSIL+ risk of only 3.4%. This finding suggests that a longer follow-up interval could be justified, streamlining screening resources. In Finland, where all LSIL+ cases are currently referred to colposcopy, our data showed wide risk variation: the least oncogenic group with LSIL+ had an HSIL+ risk of 21.1%, which is lower than the 23.9% risk of the HPV16 NILM/ASC-US group [8, 23]. As birth cohorts with high coverage from the national school-based HPV vaccination program reach the screening entry age of 30, the absolute prevalence of vaccine-targeted types will fall precipitously. Recent national register data indicate that 80% of girls and 74% of boys born in 2012 have received at least one vaccine dose, with similar trends reflected in the Tampere region [24]. This shifts the clinical focus directly onto the relative risks of the remaining non-vaccine HR-HPV types.

Extended HPV genotyping can identify women at particularly high risk, such as those with HPV16 infection, who may benefit from immediate colposcopy even in the presence of NILM/ASC-US cytology. In contrast, infections with the lowest-risk genotypes (HPV59/39/68/51/56/66) were associated with very low HSIL+ incidence, suggesting that longer follow-up intervals may be appropriate for these women and could help optimize screening resources. The marked disparity in risk across HPV genotypes supports risk-based triage strategies that incorporate both HPV genotype and cytology, rather than relying solely on the uniform LSIL+ cytology referral threshold currently used, e.g. in Finland’s program [8, 23, 25].

The varying progression risk associated with individual HPV genotypes warrants further investigation to optimize cervical cancer screening algorithms. In particular, studies evaluating HPV persistence and viral sublineage variation may help explain differences in HSIL+ risk across populations. In vaccinated populations, additional research is needed to assess the long-term cancer risk and clinical outcomes associated with non-vaccine HPV types. Furthermore, future studies should examine alternative follow-up protocols for HPV18/45 infections given their distinct carcinogenic pathway.

The primary strength of this study is the use of real-world data from an established national cervical cancer screening program, which ensures broad clinical relevance and direct applicability to clinical triage algorithms and guideline development. As a non-selected, population-based cohort rather than an enriched referral sample, these risk estimates carry strong external validity and generalizability to other high-income countries operating organized, registry-driven primary HPV screening programs. The large sample size allowed for precise estimations of HSIL+ risks for individual HR-HPV genotypes, including stratification by cytological outcomes. A limitation of our study involves the exclusion of 210 samples that tested negative or exclusively low-risk positive upon reevaluation; rather than being solely due to sample degradation during storage, this discrepancy likely reflects well-documented variations in analytical sensitivity and cutoff thresholds between different commercial testing platforms. Furthermore, the use of histology confirmed HSIL+ (CIN2+) as the endpoint, rather than invasive cervical cancer, means conclusions about actual cancer risk must be drawn alongside studies focusing on cancer tissue analysis [21]. The lack of persistence data for the HR-HPV genotypes was also a limitation [26, 27].

Our findings provide robust, population-based genotype-specific prevalence and HSIL+ risk estimates for the 14 HR-HPV genotypes individually. These findings highlight the value of extended genotyping triage in cervical cancer screening and suggest that incorporating such genotyping, including possible age-stratification, into the Finnish national screening algorithms could improve clinical management by targeting more intensive follow-up for those with the highest risk.

## Supplementary Material

ckag139_Supplementary_Data

## Data Availability

Individual participant data that underlie the results reported in this article, after de-identification, will be available for researchers who provide a methodologically sound proposal with achievable aims. Proposals should be directed via e-mail to the corresponding author (K.L.); data requestors will need to sign a data access agreement. Key points Extended genotyping demonstrated a clear hierarchy of high-risk HPV risk disparity for detection of cervical precancer. Implementing genotype-specific risk thresholds is strongly supported for optimizing national screening algorithms, allowing better identification of high-risk cases and reducing unnecessary referrals. Using real-world data from the Finnish national screening program, we provide genotype-specific prevalence and cumulative HSIL+ risk estimates, including reflex cytology outcomes, for 14 high-risk HPV types. Unlike many prior studies conducted in clinical trials or referral settings, our findings reflect routine screening performance and offer directly applicable risk estimates for population-level risk stratification and triage algorithm development.
