# Supplementary material for: Improving cervical screening risk stratification through extended human papillomavirus genotyping and age-specific data
Source: Eur J Public Health. 2026 Aug 17;36(5):ckag139. doi: 10.1093/eurpub/ckag139 (PMC13479743; doi:10.1093/eurpub/ckag139)
Supplement: Supplementary file 1 [file ckag139_supplementary_data.docx]

| **Supplementary Table 1.** Cumulative incidence of histology confirmed HSIL+ of the 14 HR-HPV genotypes accounting only single HPV infections, stratified by age groups of 30-35, 40-45 and 50-60. | | | | | | |
| --- | --- | --- | --- | --- | --- | --- |
|  |  | 30–35  (n=839) | 40–45  (n=397) | 50–60  (n=574) | All  (n=1810) |  |
| HR-HPV single infection genotype | n | Cumulative HSIL+ % | Cumulative HSIL+ % | Cumulative HSIL+ % | Cumulative HSIL+ % | P-values* |
| HPV16 | 294 | 40.00 | 32.26 | 20.90 | 34.01 | 0.020 |
| HPV18 | 109 | 19.30 | 36.36 | 6.67 | 19.27 | 0.027 |
| HPV31 | 219 | 20.18 | 17.50 | 5.71 | 15.07 | 0.027 |
| HPV33 | 87 | 29.27 | 23.53 | 6.90 | 20.69 | 0.071 |
| HPV35 | 59 | 12.50 | 27.27 | 12.50 | 15.25 | 0.470 |
| HPV39 | 97 | … | 8.00 | 9.09 | 4.12 | 0.107 |
| HPV45 | 160 | 7.81 | 14.71 | 6.45 | 8.75 | 0.370 |
| HPV51 | 133 | 7.58 | … | 6.82 | 6.02 | 0.405 |
| HPV52 | 182 | 17.44 | 9.09 | 9.62 | 13.19 | 0.275 |
| HPV56 | 115 | 5.26 | 9.38 | … | 4.35 | 0.131 |
| HPV58 | 79 | 25.81 | 23.81 | 14.81 | 21.52 | 0.571 |
| HPV59 | 81 | 4.76 | … | 4.55 | 3.70 | 0.661 |
| HPV66 | 126 | 2.17 | 14.29 | 3.85 | 5.56 | 0.069 |
| HPV68 | 69 | 3.57 | 9.52 | … | 4.35 | 0.316 |
| Any HR-HPV | 1810 | 18.12 | 16.88 | 8.19 | 14.70 | 0.000 |
| P-values** |  | 0.000 | 0.002 | 0.122 | 0.000 |  |
| *P-values for the difference in cumulative incidence between different age groups  ** P-values for the difference in cumulative incidence between the 14 high-risk HPV genotypes | | | | | | |
